# ‘Multi-SpaM’: a maximum-likelihood approach to phylogeny reconstruction using multiple spaced-word matches and quartet trees

**DOI:** 10.1093/nargab/lqz013

**Published:** 2019-10-30

**Authors:** Thomas Dencker, Chris-André Leimeister, Michael Gerth, Christoph Bleidorn, Sagi Snir, Burkhard Morgenstern

**Affiliations:** 1 Department of Bioinformatics, Institute of Microbiology and Genetics, Universität Göttingen, Goldschmidtstr. 1, 37077 Göttingen, Germany; 2 Institute for Integrative Biology, University of Liverpool, Biosciences Building, Crown Street, L69 7ZB Liverpool, UK; 3 Department of Animal Evolution and Biodiversity, Universität Göttingen, Untere Karspüle 2, 37073 Göttingen, Germany; 4 Museo Nacional de Ciencias Naturales, Spanish National Research Council (CSIC), 28006 Madrid, Spain; 5 Institute of Evolution, Department of Evolutionary and Environmental Biology, University of Haifa, 199 Aba Khoushy Ave. Mount Carmel, Haifa, Israel; 6 Göttingen Center of Molecular Biosciences (GZMB), Justus-von-Liebig-Weg 11, 37077 Göttingen, Germany

## Abstract

Word-based or ‘alignment-free’ methods for phylogeny inference have become popular in recent years. These methods are much faster than traditional, alignment-based approaches, but they are generally less accurate. Most alignment-free methods calculate ‘pairwise’ distances between nucleic-acid or protein sequences; these distance values can then be used as input for tree-reconstruction programs such as neighbor-joining. In this paper, we propose the first word-based phylogeny approach that is based on ‘multiple’ sequence comparison and ‘maximum likelihood’. Our algorithm first samples small, gap-free alignments involving four taxa each. For each of these alignments, it then calculates a quartet tree and, finally, the program ‘Quartet MaxCut’ is used to infer a super tree for the full set of input taxa from the calculated quartet trees. Experimental results show that trees produced with our approach are of high quality.

## INTRODUCTION

Sequence-based phylogeny reconstruction is a fundamental task in computational biology. Standard phylogeny methods rely on ‘sequence alignments’ of either entire genomes or sets of orthologous genes or proteins. ‘Character-based’ methods such as ‘Maximum Parsimony’ (1,2) or ‘Maximum Likelihood’ (3) infer trees based on evolutionary substitution events that may have happened since the species evolved from their last common ancestor. These methods are generally considered to be accurate as long as the underlying alignment is of high quality and as long as suitable substitution models are used. However, for the task of multiple alignment no exact polynomial-time algorithm exists, and even heuristic approaches are relatively time consuming (4). Similarly, exact algorithms for character-based approaches are known to be ‘NP hard’ (5,6).

‘Distance’ methods, by contrast, infer phylogenies by estimating evolutionary distances for all pairs of input taxa. Here, pairwise alignments are sufficient and can be faster calculated than multiple alignments, but still require run time proportional to the product of the lengths of the aligned sequences. However, there is a loss in accuracy compared to character-based approaches, as all information about evolutionary events is reduced to a single number for each pair of taxa, and not more than two sequences are considered simultaneously, as opposed to character-based approaches, where all sequences are examined simultaneously. The final trees are obtained by clustering based on the distance matrices, most commonly with ‘Neighbor Joining (NJ)’ (7) or ‘BIONJ’ (8). Since both pairwise and multiple sequence alignment is computationally expensive, they are ill-suited for the increasingly large data sets that are available today due to the next-generation sequencing techniques.

In recent years, a large number of fast ‘alignment-free’ methods have been proposed for phylogeny reconstruction, see (9–15) for review articles. Some of these approaches are using some sort of ‘micro-alignments’ and infer phylogenetic distances from the number of mismatches in these simplified alignments. So, strictly-spoken, these methods are not ‘alignment-free’, but most authors refer to them as ‘alignment-free’ anyway, since ‘micro-alignments’ can be found by rapid pattern-matching algorithms, avoiding the need to calculate full alignments of the compared sequences.

Another advantage of the so-called ‘alignment-free’ methods for genome comparison is that they can circumvent common problems of alignment-based approaches such as genome rearrangements and duplications. Moreover, many alignment-free methods can be applied not only to entire genomes, but also to partially sequenced genomes or even to unassembled reads (16–22). A disadvantage of these methods is that they are often considerably less accurate than slower, alignment-based methods. A systematic evaluation of existing alignment-free methods for a variety of different application scenarios has been carried out in the ‘AFproject’ (23).

‘co-phylog’ (18) is a recently proposed ‘alignment-free’ method that is based on ‘micro alignments’. This approach finds short, gap-free alignments of a fixed length, consisting of matching nucleotide pairs only—except for the middle position in each alignment, where mismatches are allowed. Phylogenetic distances are estimated from the fraction of such alignments for which the middle position is a mismatch. As a generalization of this approach, ‘andi’ (24) uses pairs of maximal exact word matches that have the same distance to each other in both sequences and uses the frequency of mismatches in the segments between those matches to estimate the number of substitutions per position between two input sequences. A further development of this approach is ‘phylonium’ (25).

Since ‘co-phylog’ and ‘andi’ require a minimum length of the flanking word matches in order to reduce the number of matches that are mere random background matches, they do not perform well on distantly related sequences where fewer exact matches with the required minimum length can be found, if any at all. Moreover, the number of random segment matches grows quadratically with the length of the input sequences while the expected number of homologous matches grows only linearly. Thus, the minimum match length must be increased in these approaches if long sequences are to be compared to limit the number of background matches. This, in turn, reduces the number of homologous segment matches that are found, and therefore the amount of information that is available to estimate phylogenetic distances.

Other alignment-free approaches are based on the length of maximal common substrings between sequences that can be rapidly found using suffix trees or related data structures (26,27). As a generalization of this approach, some methods use longest common substrings with a certain number of mismatches (28–32). Finally, methods have been proposed that estimate phylogenetic distances from the decay of the number of word matches as a function of the word length (33,34).

**Figure 1.**
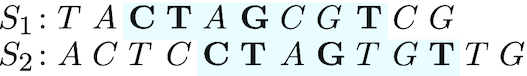
Spaced-word match *W* with respect to a pattern *P* = 1101001 of weight $w$ = 4. *W* can be seen as a gap-free pairwise alignment that has the same length as *P*, with matching nucleotide at the four ‘match positions’ and possible mismatches at the three ‘don’t-care’ positions.

In previous publications, we proposed to use words with ‘wildcard characters’—so-called ‘spaced words’—for alignment-free sequence comparison (35–37). Here, a binary pattern of ‘match’ and ‘don’t-care’ positions specifies the positions of the ‘wildcard’ characters, see also (38–40). In ‘Filtered Spaced-Word Matches (FSWM)’ (41) and ‘Proteome-based Spaced-Word Matches (Prot-SpaM)’ (42), alignments of such spaced words are used where sequence positions must match at the ‘match’ positions while mismatches are allowed at the ‘don’t-care positions’, see Figure 1. A score is calculated for every such spaced-word match in order to remove—or ‘filter out’—‘background’ spaced-word matches; the mismatch frequency of the remaining ‘homologous’ spaced-word matches is then used to estimate the number of substitutions per position that happened since two sequences evolved from their last common ancestor. The filtering step allows us to use patterns with fewer match positions in comparison to above mentioned methods ‘co-phylog’ and ‘andi’, since the vast majority of the background noise can be eliminated reliably. Consequently, phylogenetic distances calculated with ‘FSWM’ and ‘Prot-SpaM’ are still accurate, if large and distantly related sequences are compared.

**Figure 2.**
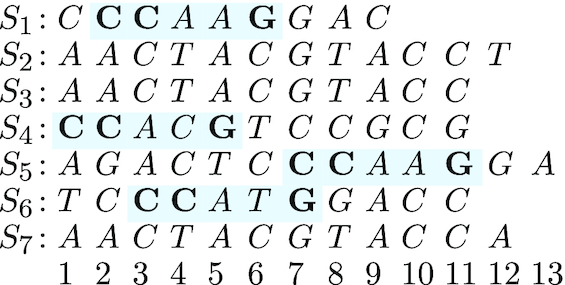
*P*-block for a pattern *P* = 11001: the spaced word *W* = CC**G occurs at [*S*_1_, 2], [*S*_4_, 1], [*S*_5_, 7] and [*S*_6_, 3].

In the present paper, we introduce a novel approach to phylogeny reconstruction called ‘Multiple Spaced-Word Matches (Multi-SpaM)’ that combines the ‘speed’ of the so-called ‘alignment-free’ methods with the ‘accuracy’ of the ‘Maximum-Likelihood’ approach. While other alignment-free methods are limited to ‘pairwise’ sequence comparison, we generalize our previous ‘FSWM’ approach to ‘multiple’ sequence comparison. For a binary pattern *P* representing ‘match’ and ‘don’t-care’ positions, ‘Multi-SpaM’ identifies so-called ‘P-blocks’ consisting of four matching spaced words from four different sequences each. That is, a *P*-block can be seen as a gap-free ‘micro alignment’ of four different sequences, with matching nucleotides at the ‘match’ positions of the underlying binary pattern and possible mismatches at its ‘don’t-care’ positions, see Figure 2 for an example. For each such *P*-block, an optimal ‘Maximum-Likelihood’ tree topology is calculated with the software ‘RAxML’ (43). We then use the ‘Quartet MaxCut’ algorithm (44) to obtain a super tree from the calculated quartet tree topologies. We show that on both simulated and real data, ‘Multi-SpaM’ produces phylogenetic trees of high quality and often outperforms other alignment-free methods. An earlier version of the present paper has been published in the proceedings of the ‘RECOMB-CG’ conference (45).

## MATERIALS AND METHODS

### Spaced words and *P*-blocks

To describe ‘Multi-SpaM’, we first need to introduce some formal definitions. We want to compare sequences over an alphabet ${\cal A}$; since our approach is dealing with DNA sequences, our alphabet is ${\cal A} = \lbrace A,C,G,T\rbrace$. For a pattern length ℓ and a binary pattern *P* ∈ {0, 1}^ℓ^, a ‘spaced word’ with respect to *P* is a word *W* of length ℓ over ${\cal A}\cup \lbrace *\rbrace$, such that *W*(*i*) = * if and only if *P*(*i*) = 0. A spaced word *W* can be considered as a regular expression where ‘*’ is a ‘wildcard character’. A position *i* ∈ {1, …, ℓ} is called a ‘match position’ if *P*(*i*) = 1 and a ‘don’t-care position’ otherwise. The number of match positions in *P* is called the ‘weight’ of *P*. For a DNA Sequence *S* of length *n* and a position 1 ≤ *i* ≤ *n* − ℓ + 1, we say that a ‘spaced word’ *W* with respect to *P* occurs in *S* at position *i* – or that [*S, i*] is an ‘occurrence’ of *W* – if *S*(*i* + *j* − 1) = *W*(*j*) for all match positions *j* of *P*. This corresponds to the definition previously used in (35) and (37).

A pair ([*S, i*], [*S*′, *i*′]) of occurrences of the same spaced word *W* is called a ‘spaced-word match’. For a substitution matrix assigning a ‘score’ *s*(*X, Y*) to every pair (*X, Y*) of nucleotides, we define the ‘score’ of a spaced-word match ([*S, i*], [*S*′, *i*′]) of length ℓ as

$$\begin{equation*} \sum _{1\le k \le \ell } s(S(i+k-1),S^{\prime }(i^{\prime }+k-1)) \end{equation*}$$

That is, if we align the two occurrences of *W* to each other, the score of the spaced-word match is the sum of the scores of the nucleotides aligned to each other. In ‘Multi-SpaM’, we are using the following nucleotide substitution matrix that has been proposed in (46):

(1) $$\begin{equation*} \begin{array}{crrrr}& A & C & G & T \\ A & 91 & -114 & -31 & -123 \\ C & & 100 &-125 & -31 \\ G & & & 100 & -114 \\ T & & & & 91 \\ \end{array} \end{equation*}$$

‘Multi-SpaM’ starts with generating a binary pattern *P* with user-defined length ℓ and weight $w$; by default, we use values ℓ = 110 and $w$ = 10, i.e. by default the pattern has 10 ‘match positions’ and 100 ‘don’t-care’ positions. We are using a low ‘weight’ to obtain a large number of spaced-word matches when comparing two sequences. This includes necessarily a high proportion of random spaced-word matches. The high number of ‘don’t-care’ positions, on the other hand, allows us to accurately distinguish between ‘homologous’ and ‘background’ spaced-word matches.

Given these parameters, a pattern *P* with minimal ‘overlap complexity (OC)’ is calculated by running our previously developed software tool ‘rasbhari’ (47). The OC of a pattern or a set of patterns is defined in terms of the number of overlapping 1’s if the patterns are shifted against themselves and against each other, for multiple pattern sets. It has been shown that the OC of patterns is closely related to their ‘sensitivity’ in database searching (48,49) and to the statistical stability of the number of spaced-word matches (37).

As a basis for phylogeny reconstruction, we are using four-way ‘micro alignments’ consisting of occurrences of the same spaced word with respect to *P* in four different sequences or their reverse complements. We call such an alignment a ‘quartet *P*-block’ or a *P*-block, for short. A *P*-block is, thus, a gap-free alignment of length ℓ where in the *k*-th column identical nucleotides are aligned if *k* is a ‘match’ position in *P*, while mismatches are possible if *k* is a ‘don’t-care’ position (see Figure 2). ‘Multi-SpaM’ considers *P*-blocks involving spaced words from both strands of the input sequences. It is clear that the number of *P*-blocks can be very large: if there are *n* occurrences of a spaced-word *W* in *n* different sequences, then this gives rise to $n \atopwithdelims ()4$ different *P*-blocks. Thus, instead of using all possible *P*-blocks, ‘Multi-SpaM’ randomly samples a limited number of *P*-blocks to keep the program run time under control.

For phylogeny reconstruction, we want to use *P*-blocks that are likely to represent true homologies. Therefore, we introduce the following definition: a *P*-block is called ‘homologous’ if it contains at least ‘one’ spaced-word occurrence [*S, i*], such that each of the three spaced-word matches of [*S, i*] with the remaining occurrences has a positive score. Note that a ‘homologous’ *P*-block in the sense of this formal definition is, of course, not necessarily ‘homologous’ in the usual sense, i.e. the four involved sequence segments are not necessarily derived from one common anchestral segment. To sample a list of homologous *P*-blocks in the sense of our definition, we randomly select spaced-word occurrences with respect to *P* from the input sequences and their reverse complements. For each selected [*S, i*], we then randomly select occurrences of the same spaced word from sequences $S^{\prime } \not= S$, until we have found three occurrences of *W* from three different sequences that all have positive scores with [*S, i*].

To find spaced-word matches efficiently, we first sort the list of all spaced-word occurrences with respect to *P* in lexicographic order, such that all occurrences of the same spaced word appear as a contiguous section of the list. Once we have sampled a homologous *P*-block as described, we remove the four spaced-word occurrences from our list, so no two of the sampled *P*-blocks can contain the same occurrence of a spaced word. The algorithm continues to sample *P*-blocks until no further *P*-blocks can be found, or until a maximal number *M* of *P*-blocks is reached. By default, ‘Multi-SpaM’ uses a maximum of *M* = 1 000 000 *P*-blocks, but this parameter can be adjusted by the user.

### Quartet trees

For each of the sampled quartet *P*-blocks, we infer an unrooted binary tree topology. This most basic phylogenetic unit is called a ‘quartet’ topology; there are three different quartet topologies for a set of four taxa. To identify the best of these three topologies, we use the ‘Maximum-Likelihood’ software ‘RAxML’ (43) with the ‘GTR’ model (50). This corresponds to using the command-line version of ‘RAxML’ with the option ‘-m GTRGAMMA -f q -p 12345’. We note that ‘RAxML’ is a general ‘maximum-likelihood’ software, its use in our context is fairly degenerated, as we only use it to infer optimal quartet topologies.

After obtaining the optimal quartet topology for each of the sampled *P*-blocks, we need to amalgamate them into a single tree spanning the entire taxa set. This task is called the ‘Supertree Task’ (51) and is known to be ‘NP hard’, even for the special case where the input is limited to quartets topologies, as in our case (52). Nevertheless there are several heuristics for this task, with ‘MRP’ (53,54) the most popular. Here we chose to use ‘Quartet MaxCut’ (44,55) that proved to be faster and more accurate for this kind of input (56). In brief, ‘Quartet MaxCut’ recursively partitions the taxa set, where each such partition defines a split in the final tree. If, during this process, a set *A* of taxa is to be split into two subsets, the program tries to put neighboring taxa from the quartet trees into the same subset while non-neighboring taxa can end up in different subsets. To achieve this, a multi-graph is defined where the taxa in the set *A* are represented as nodes, and each pair of taxa in each quartet tree is represented as an edge. That is, each quartet tree defines six edges in the multi-graph. Edges between neighboring taxa in a quartet tree are seen as ‘good’ edges that are to be retained, if possible, while edges between non-neighboring taxa are ‘bad’ edges that can be removed by the partition. The program then finds a partition that minimizes the ratio between good and bad edges that are to be removed, see (44,55) for details.

### Implementation

To keep the run time of our software manageable, we integrated the ‘RAxML’ code directly into our program code. We parallelized our program with ‘openmp’ (57).

## TEST RESULTS

To evaluate ‘Multi-SpaM’ and to compare it to other fast, alignment-free methods, we applied these approaches to both simulated and real sequence data and compared the resulting trees to reference trees. In phylogeny reconstruction, artificial benchmark data are often used since here, ‘correct’ reference trees are known. For the real-world sequence data that we used in our study, we had to rely on reference trees that are believed to reflect the true evolutionary history, or on trees calculated using traditional, alignment-based methods that can be considered to be reasonably accurate. In our test runs, we used standard parameters for all methods, if such parameters were suggested by the respective program authors. The program ‘kmacs’ (28) that was one of the programs that we evaluated, has no default value for its only parameter, the number *k* of allowed mismatches in common substrings. Here, we chose a value of *k* = 4. While ‘Multi-SpaM’ produces tree topologies without branch lengths, all other methods that we evaluated produce distance matrices. To generate trees from these matrices, we used ‘Neighbor-joining’ (7).

To compare the trees produced by the different alignment-free methods to the respective benchmark trees, we used the ‘Robinson-Foulds (RF)’ metric (58), a standard measure to compare how different two tree topologies are. The smaller the ‘RF’ distances between the reconstructed trees and the corresponding reference trees are, the better a method is. To calculate ‘Neighbor-joining’ trees and to calculate ‘RF’ distances between the obtained trees and the respective reference trees, we used the ‘PHYLIP’ package (59).

As explained above, both ‘FSWM’ and ‘Multi-SpaM’ rely on binary patterns of ‘match’ and ‘don’t-care’ positions; the results of these programs therefore depend on the underlying patterns. Both programs use the software ‘rasbhari’ (47) to calculate binary patterns. ‘rasbhari’ uses a probabilistic algorithm, so different program runs usually return different patterns and, as a result, different program runs with ‘FSWM’ and ‘Multi-SpaM’ may produce slightly different distance estimates, even if the same parameter values are used. To see how ‘FSWM’ and ‘Multi-SpaM’ depend on the underlying binary patterns, we ran both programs ten times on each data set. The figures in the ‘Results’ section report the ‘averageRF’-distance for each data set over the ten program runs. Error bars indicate the highest and lowest RF-distances, respectively, for the 10 program runs.

### Simulated sequences

At first, we evaluated ‘Multi-SpaM’ on data sets generated with the ‘Artificial Life Framework (ALF)’ (60). ‘ALF’ starts by simulating an ancestral genome that includes a number of genes. According to a guide tree that is either provided by the user or randomly generated, ALF simulates speciation events and other evolutionary events such as substitutions, insertions and deletions for nucleotides, as well as duplications, deletions and horizontal transfer of entire genes. A large number of parameters can be specified by the user for these events. We used parameter files that were used in a study by the authors of ALF (61). This way, we generated six data sets, three with simulated *γ*-proteobacterial genomes (*b*1, *b*2, *b*3), and three with simulated mammalian genomes (*m*1, *m*2, *m*3).

We used the base parameter sets for each data set and only slightly modified them to generate *DNA* sequences for roughly 1000 genes per taxon that we then concatenated to full genomes. As in (61), we used parameter values 7.2057 and 401.4189 for the length distribution of the simulated bacterial sequences and 1.7781 and 274.1061, respectively, for the length distribution of the simulated mammalian sequences. Within each data set, we used the same rate for gene duplication, gene loss and horizontal gene transfer, but we used different rates for different data sets. For the six data sets, the corresponding rates were set to 0.0025 (*b*1), 0.0018 (*b*2), 0.0017 (*b*3), 0.0058 (*m*1), 0.0068 (*m*2), 0.011 (*m*3), respectively. Each data set uses a different guide tree that was sampled from known topologies. The average pairwise distances in our simulated sequence sets are as follows (average number of substitutions per position as estimated with *FSWM*): *m1: 0.11; m2: 0.12; m3: 0.07; b1: 0.30; b2: 0.23; b3: 0.25*.

**Figure 3.**
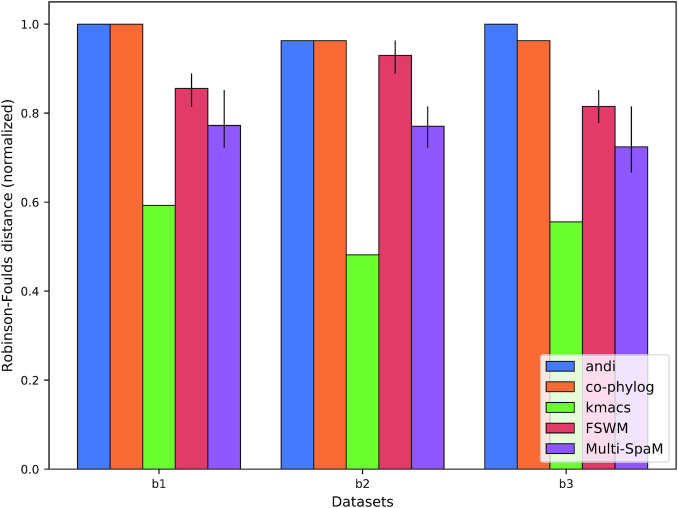
Average ‘normalized Robinson-Foulds (RF)’ distances between trees calculated with alignment-free methods and reference trees for three sets of simulated bacterial genomes. ‘FSWM’ and ‘Multi-SpaM’ were run 10 times, with different patterns *P* generated (see the main text). Error bars indicate the lowest and highest RF distances, respectively.

**Figure 4.**
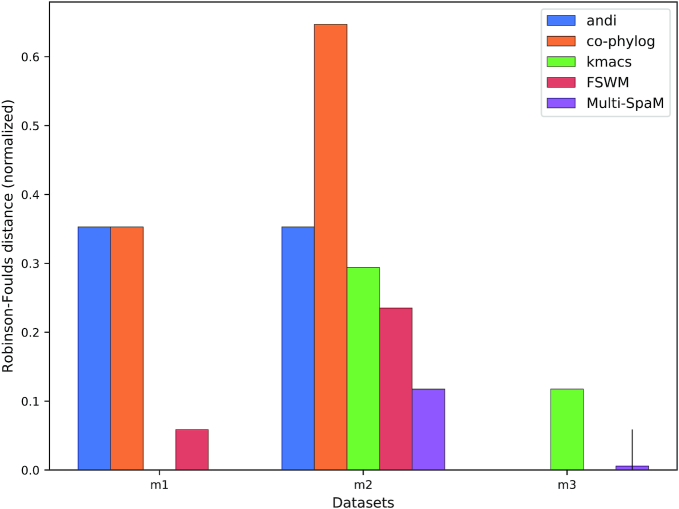
‘Normalized RF’ distances for three sets of simulated mammalian genomes. If no bar is shown, the RF distance is zero for the respective method and data set. For example, the RF distance between the tree generated by ‘kmacs’ for data set *m1* and the reference tree is zero, i.e. here the reference tree topology was precisely reconstructed. Error bars for ‘FSWM’ and ‘Multi-SpaM’ are as in Figure 3.

Each data generated in this way set contains 30 genomes (taxa) and has a size of around 10 *mb*. As shown in Figure 3, none of the tools that we evaluated was able to exactly reconstruct the reference tree topologies for the simulated bacterial genomes. In some cases, the average normalized RF distance to the reference trees was 1.0, the maximum possible dissimilarity value. Therefore, we also calculated the triplet distance between the reconstructed trees and the reference trees by running the program ‘tqDist’ (62). The results are shown in the Supplementary Data. Reference topologies for the simulated mammalian genomes could be reconstructed by some tools, although no method could reconstruct all three reference topologies exactly, see Figure 4.

We also evaluated how the parameters of the genome sequence simulator ‘ALF’ affect the performance of ‘Multi-SpaM’ on the simulated genomes. A figure showing the influence of these parameters is given in the Supplementary Data. In short, the rate of ‘horizontal gene transfer (HGT)’ has a larger influence on the quality of the resulting trees than other parameters of ‘ALF’. This is not surprising, since ‘HGT’ events can lead to false quartet tree topologies, whereas the other program parameters mostly affect the ‘number’ of *P*-blocks that can be used by ‘Multi-SpaM’, but not so much the resulting quartet topologies. Even so, the ‘HGT’ rate in ‘ALF’ had only minor influence on the quality of the resulting trees, compared to the guide tree that is used in the simulation.

### Real genomes

We also applied the programs that we evaluated to real genomes to see if the results are similar to our results on simulated genomes. Here, our first data set were 29 *Escherichia coli* and *Shigella* genomes that are commonly used as a benchmark data set to evaluate alignment-free methods (24). As a reference, we used a tree calculated with ‘Maximum Likelihood’, based on a ‘mugsy’ alignment (63). The data set is 144 *mb* large and the average distance between two sequences in this set is ∼0.0166 substitutions per sequence position.

**Figure 5.**
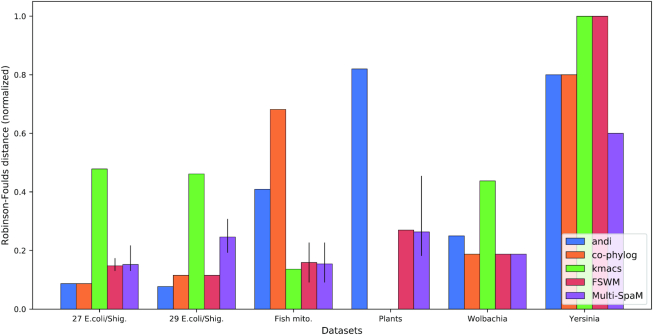
‘Normalized RF’ distances for six sets of benchmark genomes: 29 *E. coli/Shigella* genomes, another set of 27 *E. coli/Shigella* genomes, mitochondrial genomes from 25 different fish species, 14 plant genomes, 19 *Wolbachia* genomes and 8 Yersinia genomes. Error bars for ‘FSWM ’and ‘Multi-SpaM’ as in Figure 3. Unlike in Figure 4, missing bars for the plant data sets in this figure mean that the programs in question, *co-phylog* and *kmacs*, did not terminate on this data set.

Next, we used 19 *Wolbachia* genomes that have been analyzed by (64); we used the phylogeny published in their paper as a reference. The total size of this sequence set is 25 *mb*, the average pairwise distance is 0.06 substitutions per position. The results of these three series of test runs are summarized in Figure 5.

**Figure 6.**
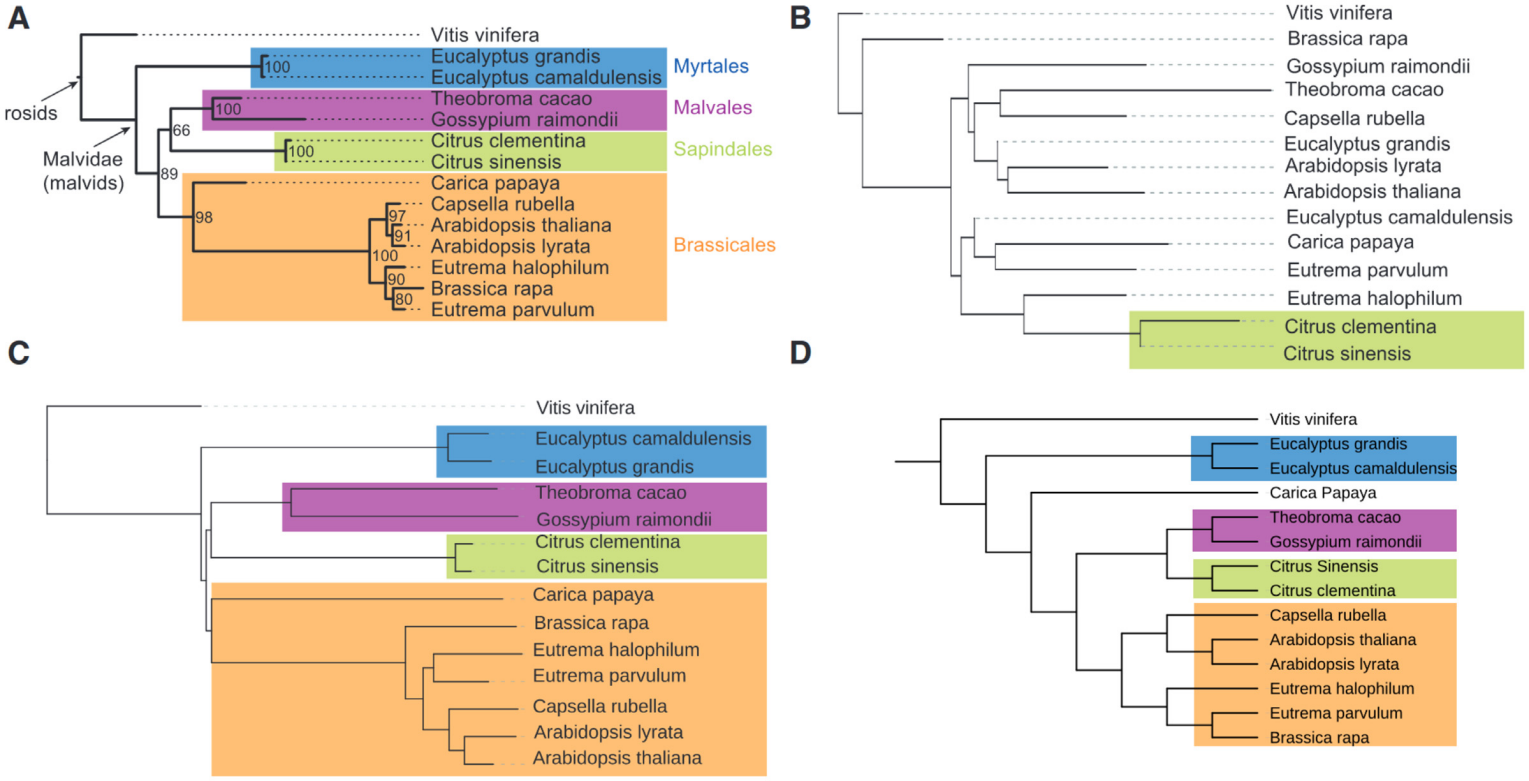
Reference tree (**A**) from (65) and trees reconstructed by ‘andi’ (**B**), ‘FSWM’ (**C**) and ‘Multi-SpaM’ (**D**) for a set of 14 plant genomes.

As a third real-world test case, we used a much larger sequence set, namely a set of 14 plant genomes with a total length of 4.8 *gb*. This data set was originally used by Hatje and Kollmar (65) and has been subsequently used as benchmark data in other publications on alignment-free methods. Figure 6 shows the resulting trees. For this data set, we used a pattern with a weight of $w$ = 12 instead of the default value $w$ = 10, to keep the number of background spaced-word matches manageable. As can be seen in Figure 6, ‘Multi-SpaM’ and ‘FSWM’ produced fairly accurate trees for this data set, with only minor differences to the reference tree: ‘Multi-SpaM’ misclassified ‘Carica papaya’, whereas ‘FSWM’ failed to classify *Brassica rapa* correctly. None of the other alignment-free tools that we evaluated could produce a reasonable tree for this data set: ‘andi’ returned a tree that is rather different to the reference tree, while ‘kmacs’ and ‘co-phylog’ could not finish the program runs in a reasonable time frame.

In addition, we used three real-world data sets that were used as benchmark data in the ‘AFproject’ paper (23): another data set of 27 *E. coli/Shigella* genomes, a set of mitochondrial genomes from 25 fish species, and a set of 8 strains of *Yersinia*.

As explained in the ’Materials and Methods’ section, ‘Multi-SpaM’ calculates an optimal tree topology for each of the sampled ‘quartet *P*-blocks’. Here, it can happen that no single best topology is found. In particular for closely related sequences, this happens for a large fraction of the sampled quartet *P*-blocks. For the *E. coli/Shigella* data set, for example, ∼50% of the *P*-blocks were inconclusive, i.e. ‘RAxML’ could find no single best tree topology. We observed a similar result for a data set of 13 *Brucella* genomes where the pairwise phylogenetic distances are even smaller than for the *E. coli/Shigella* data set, namely 0.0019 substitutions per site, on average. Here, roughly 80% of the blocks were inconclusive. For all other data sets, the fraction of inconclusive quartet *P*-blocks was negligible. For example, for the set of 14 plant genomes, only ∼250 out of the 1 000 000 sampled *P*-blocks were inconclusive.

### Program run time and memory usage

Table 1 shows the program run time for ‘Multi-SpaM’, ‘FSWM’, ‘kmacs’, ‘andi’ and ‘co-phylog’ on all six real-world data sets in our program comparison. The test runs were done on a 5 x Intel(R) Xeon(R) CPU E7-4850 with 2.00 GHz, a total of 40 threads (20 cores). Some of the programs that we evaluated have been parallelized. For these programs, both ‘wall clock time’ and ‘CPU’ time are reported. For the largest data set in our study, the set of 14 plant genomes, the peak ‘RAM' usage was 76 GB for ‘FSWM’, 110 GB for ‘andi’ and 142 GB for ‘Multi-SpaM’.

**Table 1.** Run time in seconds for different alignment-free approaches on six sets of real-world genomes. On the largest data set, the 14 plant genomes, ‘kmacs’ and ‘co-phylog’ did not terminate the program run. On this data set, we increased the pattern weight for ‘Multi-SpaM’ from the default value of $w$ = 10 to $w$ = 12, in order to reduce the run time. Note that ‘Multi-SpaM’, ‘FSWM’ and ‘andi’ are parallelized, so we could run them on multiple processors, while ‘kmacs’ and ‘co-phylog’ had to be run on single processors. The reported run times are ‘wall clock times’.

	FSWM		andi		co-phylog	kmacs	Multi-SpaM	
	wall clock	CPU	wall clock	CPU			wall clock	CPU
27 *E.coli/Shigella*	710	27,075	15	291	704	5,247	603	22,185
29 *E.coli/Shigella*	860	32,798	16	325	533	55,736	611	21,973
25 fish mitochondria	2	8	<1	3	9	5	27	1,054
14 plants	1,107,720	28,690,489	1,808	13813	-	-	12,516	389,770
19 *Wolbachia*	65	2,185	3	42	113	24,961	484	15,804
8 *Yersinia*	91	3,333	5	34	50	1,083	183	6,182

In memory saving mode, the peak ‘RAM’ usage of ‘Multi-SpaM’ could be reduced to 10.5 *GB*, but this roughly doubles the program run time. To achieve this, the list of spaced words is not kept in memory in its entirety, but rather in 16 chunks based on the first two match positions. At any given time, there is only one chunk kept in main memory in addition to the sequences itself and the list of *P*-blocks. The overhead, such as additional comparisons, results in increased run times.

## DISCUSSION

Standard software tools for phylogeny reconstruction are relatively slow, because they rely on multiple sequence alignments and on time-consuming probabilistic calculations. Therefore, a variety of so-called ‘alignment-free’ methods have been proposed recently, which are orders of magnitudes faster than those alignment-based approaches. Existing alignment-free methods calculate ‘distances’ between DNA or protein sequences that can be used as a basis for phylogeny reconstruction. In general, however, distance-based phylogeny methods are considered to be less accurate than ‘character-based’ methods. In this paper, we introduced a novel approach to phylogeny reconstruction called ‘Multi-SpaM’ that combines the speed of alignment-free methods with the accuracy of ‘Maximum Likelihood’. To our knowledge, this is the first alignment-free approach that uses multiple sequence comparison and likelihood.

Our test runs show that ‘Multi-SpaM’ can produce phylogenetic trees of high quality. It outperforms other alignment-free methods on a number of test data sets, in particular on sequences with large evolutionary distances. On sets of very similar sequences, such as different strains of the same bacterial species, however, our approach was sometimes outperformed by other alignment-free methods. As shown in Figure 5, the programs ‘andi’, ‘co-phylog’ and ‘FSWM’ produce better results than ‘Multi-SpaM’ on a set of *E. coli/Shigella* genomes. This may be due to our above-mentioned observation that there is often no single best tree topology for a ‘quartet *P*-block’, if the compared sequences are very similar to each other.

As mentioned in the ‘Results’ section, we used ‘Neighbor-Joining (NJ)’ (7) in order to obtain phylogenetic trees from the distance matrices produced by the competing alignment-free programs ‘andi’, ‘co-phylog’, ‘FSWM’ and ‘kmacs’. As an alternative, we also ran the program ‘BIONJ’ (8). It should be mentioned that, in the majority of test runs, ‘*BIONJ’*’ produced slightly better results than ‘NJ’, especially on the distance matrices produced by ‘FSWM’. In our program evaluation, we used ‘NJ’ anyway, since this program is used in most other studies to evaluate alignment-free methods, e.g. in the recently published ‘AFproject’ benchmark study (23), so using ‘NJ’ makes it easier to compare our results to other studies.

Calculating optimal tree topologies for the sampled ‘quartet *P*-blocks’ is a relatively time-consuming step in ‘Multi-SpaM’. In fact we observed that, for a given set of input sequences, the program run time of ‘Multi-SpaM’ is roughly proportional to the number of *P*-blocks for which topologies are calculated. However, the maximal number of ‘quartet blocks’ that are sampled is a user-defined parameter. By default we sample up to *M* = 1 000 000 quartet blocks; in our test runs, the quality of the resulting trees could not be significantly improved by further increasing *M* (test results with different values of *M* are shown in the Supplementary Data). Consequently, our method is relatively fast on large data sets, where only a small fraction of the possible quartet-blocks is sampled. By contrast, on small data sets, ‘Multi-SpaM’ is slower than other alignment-free methods. To further speed-up ‘Multi-SpaM’, we have parallelized our software to run on multiple cores; in Table 1, we report both wall-clock and ‘CPU’ run times. It should be straight-forward to adapt our software to run on distributed systems, as has been done for other alignment-free approaches (66,67).

Apart from the maximum number of sampled quartet blocks, the only relevant parameters of our approach are the ‘length’ and the ‘weight’ (number of ‘match positions’) of the underlying binary pattern. For ‘Multi-SpaM’, we used similar default values as in ‘Filtered Spaced Word Matches (FSWM)’ (41), namely a weight of $w$ = 10 and a pattern length of ℓ = 110, so our default patterns have 100 ‘don’t-care’ positions. As mentioned in the ‘Materials and Methods’ section, a large number of ‘don’t-care’ positions is important in ‘Multi-SpaM’ as well as in our previous approach ‘FSWM’, since this makes it easier to distinguish homologous from random background spaced-word matches. Also, a large number of ‘don’t-care’ positions helps to reduce the number of ‘inconclusive’ quartet *P*-blocks, where no single best quartet tree exists, on data sets where sequences are closely related to each other.

Our default pattern length ℓ = 110 limits, on the other hand, the number of homologous quartet blocks that can be found. Since ‘Multi-SpaM’ is based on ‘gap-free’ four-way alignments of length ℓ, *P*-blocks with positive scores can only be expected in sequence regions without insertions or deletions. For real-world data sets, it is difficult to tell how exactly the number of possible homologous *P*-blocks depends on the pattern length—to find out, one would need either a reliable multiple alignment of the sequences or the full list of homologous *P*-blocks. But both are impossible to calculate for large genome sequences. As a proxy, to get an idea how the number of homologous ‘quartet’ *P*-blocks is affected by the pattern length ℓ, we counted the number of ‘pairwise’ spaced-word matches with positive scores for different patterns with a fixed weight and variable length. For various real-world genomes, we found that, with our default length ℓ = 110, the number of spaced-word matches with positive scores is only slightly smaller than with a pattern length of, for example, ℓ = 60. Details are shown in the Supplementary Data.

In ‘Multi-SpaM’, we are using by default a relatively low ‘weight’ of the underlying pattern *P*, to obtain a sufficiently large number of *P*-blocks. On very large data sets, on the other hand, it is advisable to increase the weight of *P*, in order to reduce the number of the spaced-word matches that are considered, and thereby the program run time. For the largest data set our study, the set of plant genomes, we increased the pattern weight in our test runs from the default value $w$ = 10 to $w$ = 12. A table in the Supplementary Data shows that increasing the pattern weight can slightly deteriorate the quality of the resulting trees, so one should be careful with this option.

We should mention that it is, in general, not possible to predict the run time of ‘Multi-SpaM’ from the program parameters and the size of the input data alone. A relatively time-consuming step of our algorithm is sampling homologous *P*-blocks. As detailed above, this is done by iteratively picking a random spaced-word occurrence, and by looking at other random occurrences of the same spaced word in different sequences, until three spaced-word matches with positive scores are found, i.e. until a homologous *P*-block is found. Since we are using patterns with a low weight, most random spaced-word matches have negative scores. The number of spaced-word matches that have to be evaluated, until a homologous *P*-block is found, depends on the input sequences and can vary considerably. This may be the reason why the relative run time of ‘Multi-SpaM’, compared to other methods, is rather variable, as can be seen in Table 1. The instability of the program run time is a certain disadvantage of our approach.

To distinguish between homologous and background spaced-word matches, we are using a nucleotide substitution matrix that has been published by Webb Miller’s group (46), the same matrix that we are using in ‘Filtered Spaced Word Matches’ (41). As we have shown in this previous paper, homologous and background spaced-word matches can be easily distinguished if the number of ‘don’t-care positions’ is sufficiently large. The performance of our program is, thus, hardly affected by the specific substitution matrix that we are using; on most sequence sets one can expect to obtain similar results with an alternative matrix, or even by simply counting matches and mismatches.

To calculate supertrees from quartet tree topologies, the current implementation of ‘Multi-SpaM’ uses the previously developed software ‘Quartet MaxCut’ (44,55). We are using this program since it is faster and produced better results on our data than other supertree approaches. A drawback of this approach is that the current version of ‘Multi-SpaM’ generates tree ‘topologies’ only, i.e. trees without branch lengths. We will investigate in the future, if our approach can be extended to calculate full phylogenetic trees with branch lengths, based on the same ‘quartet’ *P*-blocks that we have used in the present study.

## DATA AVAILABILITY

The source code of the program is available at https://github.com/tdencker/multi-SpaM.

**Contact:** thomas.dencker@stud.uni-goettingen.de

## Supplementary Material

lqz013_Supplemental_File
