# Supplementary material for: ‘Multi-SpaM’: a maximum-likelihood approach to phylogeny reconstruction using multiple spaced-word matches and quartet trees
Source: NAR Genom Bioinform. 2019 Oct 30;2(1):lqz013. doi: 10.1093/nargab/lqz013 (PMC7671388; doi:10.1093/nargab/lqz013)
Supplement: lqz013_Supplemental_File [file lqz013_supplemental_file.pdf]

Supplementary Material for *Multi-SpaM: a Maximum-Likelihood approach to Phylogeny reconstruction using Multiple Spaced-Word Matches and Quartet Trees*

Thomas Dencker, Chris-André Leimeister, Michael Gerth, Christoph Bleidorn, Sagi Snir, Burkhard Morgenstern

July 31, 2019

In our paper, we describe *Multi-SpaM*, a new alignment-free approach to phylogeny reconstruction. For a given binary pattern P of length ℓ, consisting of w *match positions* and $\ell - w$ *don't-care positions*, our approach uses *quartet blocks*, defined as local, gap-free alignments of four different input sequences each, with identical nucleotides at the *match positions* of P and possible mismatches at the *don't-care positions*. w is called the *weight* of P. By default, we sample up to $M = 1,000,000$ *quartet blocks*. The default pattern length is $\ell = 110$, the default number of *match positions* is $w = 10$, i.e. we have 100 *don't-care* positions with these default parameter values. In the following, we show the influence of the values of M, ℓ and w on the results produced by *Multi-SpaM*.

Because *Multi-SpaM* uses gap-free alignments, one may think that the length ℓ of these alignments might affect the output of our approach: since we can only consider homologies between *indel-free* segments of the sequences, a long pattern length ℓ reduces the number of possible homologous quartet blocks that can be used by *Multi-SpaM*. It would thus be interesting to know how the total number of homologous quartet blocks depends on the pattern length. Note that the number of *quartet blocks* can be very large so, in

weight	min. RF distance	avg. RF distance	max. RF distance
10	0.130	0.152	0.217
12	0.154	0.238	0.308
14	0.154	0.254	0.346
16	0.192	0.242	0.308

Table S1: Normalized Robinson-Foulds distances for different weights on the 29 *E. coli/Shigella* dataset

general, we cannot determine this number experimentally. As a proxy, we report the number of *pairwise* spaced-word matches. Figure 1 shows, how the number of pairwise spaced-word matches with positive scores depends on the length ℓ of the underlying pattern. As can be seen, the number of spaced-word matches decreases with an increased pattern length ℓ, but the decrease is rather moderate.

Table 1 shows the influence of the *weight* (number of *match positions*) of the underlying pattern P on the quality of the trees produced with *Multi-SpaM*, as measured by their *Robinson-Foulds* distances to reference trees. Similarly, Figure 2 shows the influence of the maximal number M of quartet blocks on the quality of the reconstructed trees, while Figure 4, shows how the length of the pattern P affects the quality of the trees.

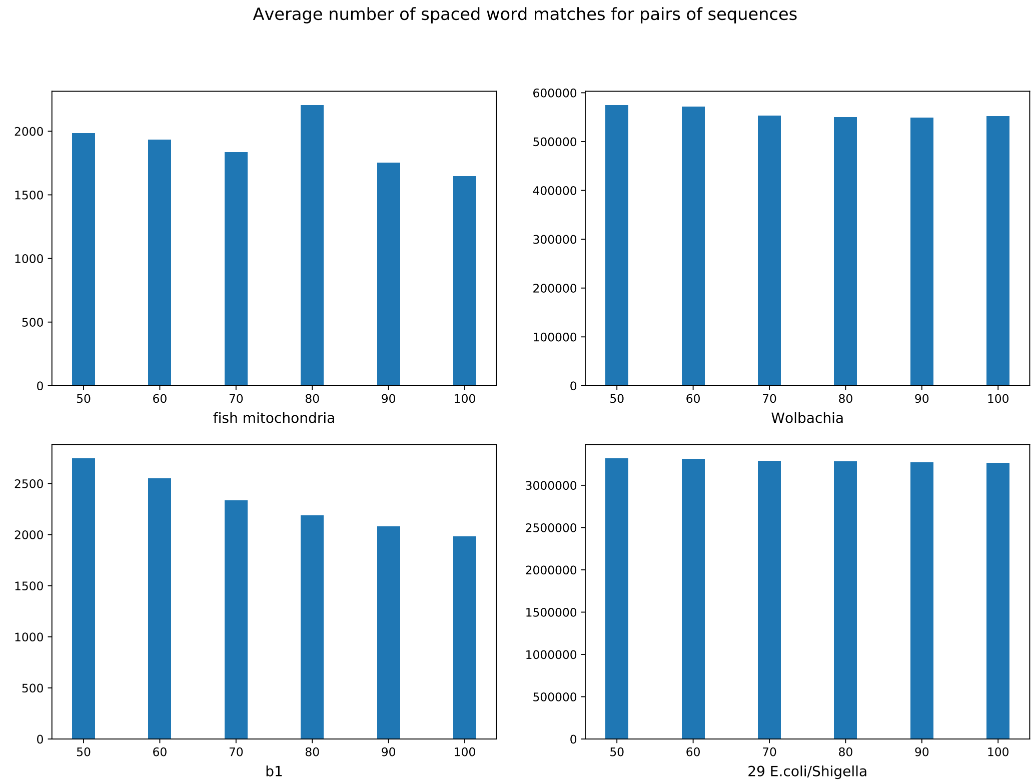

Figure S1: Number of *spaced-word* matches with positive score between different pairs of genomes for patterns P of weight $w = 10$, i.e. with 10 *match positions*, and with varying length. The x axis is the number of *don't-care* positions $\ell - w$.

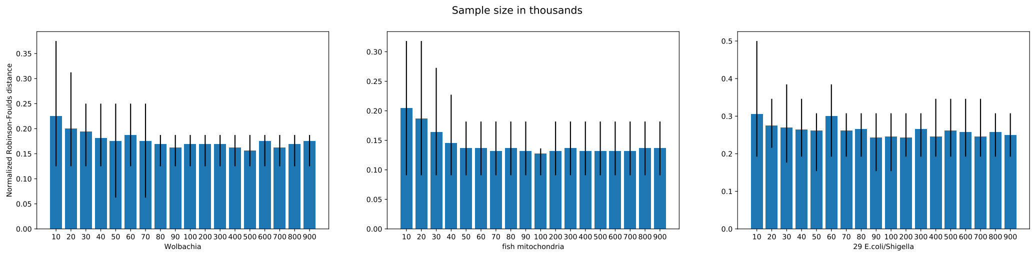

Figure S2: Average *normalized Robinson-Foulds (RF)* distances between trees calculated with *Multi-SpaM* depending on the maximal number M of sampled quartet blocks on three different data sets used in the paper: *Wolbachia*, fish mitochondrial genomes and *E. coli/shigella*. All other parameter values are default values.

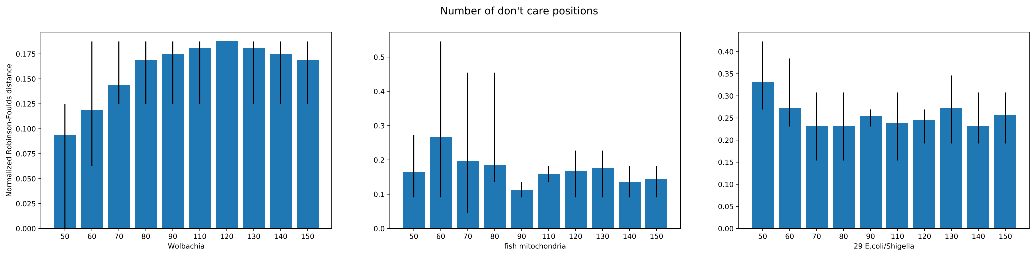

Figure S3: Average *normalized Robinson-Foulds (RF)* distances between trees calculated with *Multi-SpaM* depending on the number of *don't care* positions. All other parameter values are default values.

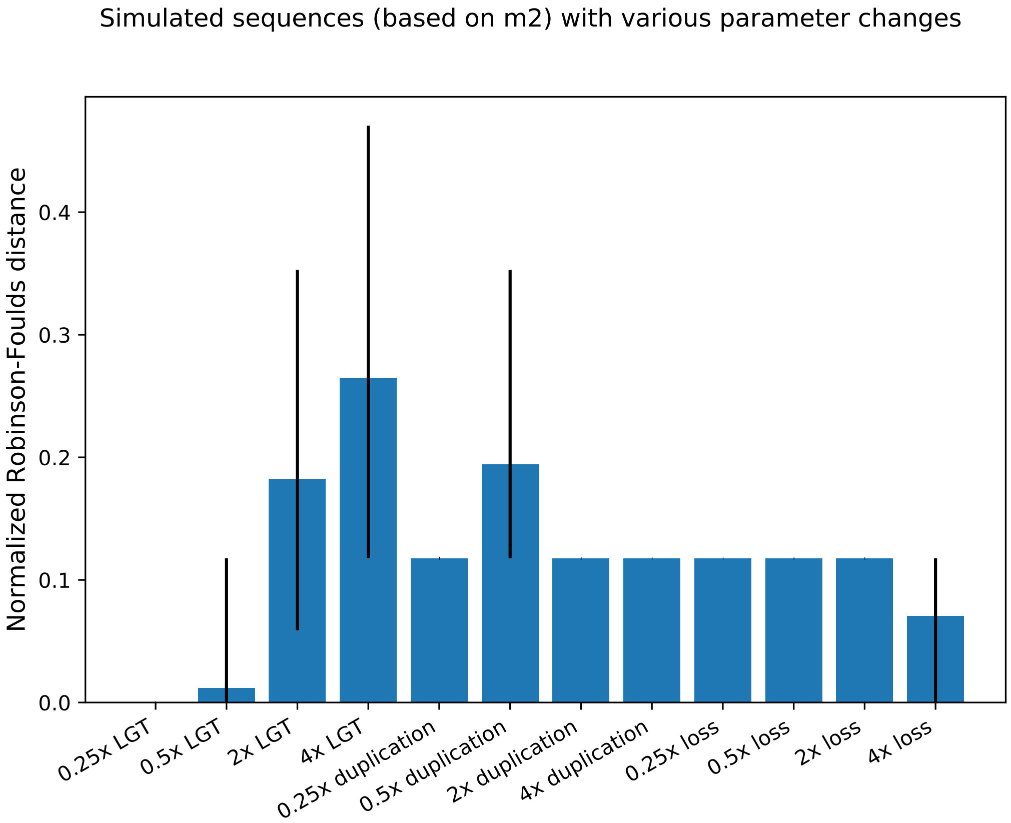

Figure S4: Average *normalized Robinson-Foulds (RF)* distances between trees calculated with *Multi-SpaM* and reference trees for different semi-artificial mammalian data sets generated with the *Artificial Life Framework (ALF)* [1]. The data sets were generated as the set *m2* described in the paper, but with different parameters for *lateral gene transfer*, gene *duplications* and *gene loss*.

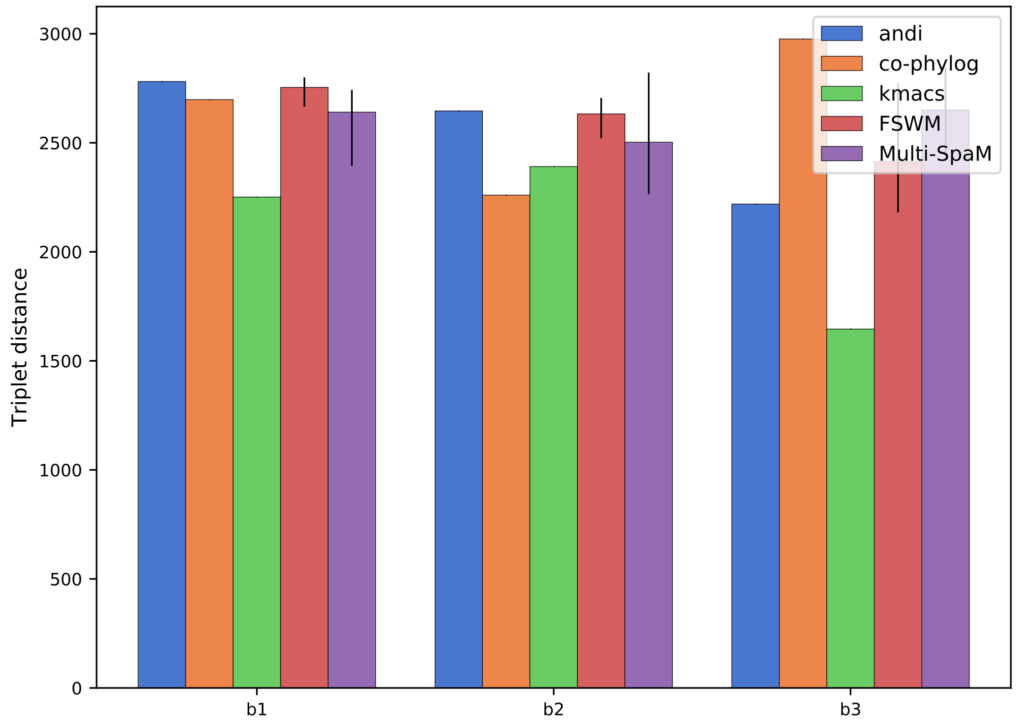

Figure S5: Average *triplet distances* calculated with *tqDist* [2] between trees calculated with alignment-free methods and reference trees for three sets of simulated bacterial genomes. Sequences were generated with the *Artificial Life Framework (ALF)* [1], see also Figure 3 in the main paper. *FSWM* and *Multi-SpaM* were run 10 times, with different patterns P generated. Error bars indicate the lowest and highest *triplet distances*, respectively.

References

[1] Daniel A. Dalquen, Maria Anisimova, Gaston H. Gonnet, and Christophe Dessimoz. ALF - a simulation framework for genome evolution. *Molecular Biology and Evolution*, 29:1115–1123, 2012.

[2] Andreas Sand, Morten K. Holt, Jens Johansen, Gerth Stlting Brodal, Thomas Mailund, and Christian N. S. Pedersen. tqDist: a library for computing the quartet and triplet distances between binary or general trees. *Bioinformatics*, 30:2079–2080, 2014.
